# Low Concentration of Withaferin a Inhibits Oxidative Stress-Mediated Migration and Invasion in Oral Cancer Cells

**DOI:** 10.3390/biom10050777

**Published:** 2020-05-17

**Authors:** Tzu-Jung Yu, Jen-Yang Tang, Fu Ou-Yang, Yen-Yun Wang, Shyng-Shiou F. Yuan, Kevin Tseng, Li-Ching Lin, Hsueh-Wei Chang

**Affiliations:** 1 Graduate Institute of Medicine, College of Medicine, Kaohsiung Medical University, Kaohsiung 80708, Taiwan; u107500035@kmu.edu.tw; 2 Department of Radiation Oncology, Faculty of Medicine, College of Medicine, Kaohsiung Medical University, Kaohsiung 80708, Taiwan; reyata@kmu.edu.tw; 3 Department of Radiation Oncology, Kaohsiung Medical University Hospital, Kaohsiung 80708, Taiwan; 4 Division of Breast Surgery and Department of Surgery, Kaohsiung Medical University Hospital, Kaohsiung 80708, Taiwan; swfuon@kmu.edu.tw; 5 Center for Cancer Research, Kaohsiung Medical University, Kaohsiung 80708, Taiwan; wyy@kmu.edu.tw (Y.-Y.W.); yuanssf@kmu.edu.tw (S.-S.F.Y.); 6 School of Dentistry, College of Dental Medicine, Kaohsiung Medical University, Kaohsiung 80708, Taiwan; 7 Cancer Center, Kaohsiung Medical University Hospital, Kaohsiung 80708, Taiwan; 8 Translational Research Center, Kaohsiung Medical University Hospital, Kaohsiung 80708, Taiwan; 9 Shanghai Jiao Tong University School of Medicine, Shanghai 200025, China; kevintseng192@sjtu.edu.cn; 10 Department of Radiation Oncology, Chi-Mei Foundation Medical Center, Tainan 71004, Taiwan; 11 School of Medicine, Taipei Medical University, Taipei 11031, Taiwan; 12 Chung Hwa University Medical Technology, Tainan 71703, Taiwan; 13 Department of Medical Research, Kaohsiung Medical University Hospital, Kaohsiung 80708, Taiwan; 14 Department of Biomedical Science and Environmental Biology, College of Life Sciences, Kaohsiung Medical University, Kaohsiung 80708, Taiwan

**Keywords:** Withaferin A, oral cancer, oxidative stress, migration, invasion, matrix metalloproteinases, antioxidant signaling

## Abstract

Withaferin A (WFA) has been reported to inhibit cancer cell proliferation based on high cytotoxic concentrations. However, the low cytotoxic effect of WFA in regulating cancer cell migration is rarely investigated. The purpose of this study is to investigate the changes in migration and mechanisms of oral cancer Ca9-22 cells after low concentrations of WFA treatment. WFA under 0.5 μM at 24 h treatment shows no cytotoxicity to oral cancer Ca9-22 cells (~95% viability). Under this condition, WFA triggers reactive oxygen species (ROS) production and inhibits 2D (wound healing) and 3D cell migration (transwell) and Matrigel invasion. Mechanically, WFA inhibits matrix metalloproteinase (MMP)-2 and MMP-9 activities but induces mRNA expression for a group of antioxidant genes, such as nuclear factor, erythroid 2-like 2 (*NFE2L2*), heme oxygenase 1 (*HMOX1*), glutathione-disulfide reductase (*GSR*), and NAD(P)H quinone dehydrogenase 1 (*NQO1*)) in Ca9-22 cells. Moreover, WFA induces mild phosphorylation of the mitogen-activated protein kinase (MAPK) family, including extracellular signal-regulated kinases 1/2 (ERK1/2), c-Jun N-terminal kinase (JNK), and p38 expression. All WFA-induced changes were suppressed by the presence of ROS scavenger *N*-acetylcysteine (NAC). Therefore, these results suggest that low concentration of WFA retains potent ROS-mediated anti-migration and -invasion abilities for oral cancer cells.

## 1. Introduction

Oral cancer leads to high morbidity and mortality [1]. It invades local tissues [2] and reoccurs occasionally [3]. Local invasions are associated with metastasis, which is important to oral carcinogenesis [4]. Therefore, discovery of a drug that inhibits metastasis or local invasion is of great importance for oral cancer therapy.

Withaferin A (WFA), a triterpenoid derived from the root or leaf of the medicinal plant *Withania somnifera*, is reported to exhibit antiproliferative properties and can induce apoptosis in several types of cancers such as leukemia [5], cervical [6], pancreatic [7], breast [8], lung [9], colorectal [10], and oral [11,12] cancer cells. These anticarcinogenic effects for WFA were based on high cytotoxic concentrations.

These cytotoxic concentrations of WFA were reported to induce reactive oxygen species (ROS)-mediated apoptosis in oral [12] and colon [10] cancer cells. ROS may induce a number of reactions such as apoptosis [5,6,7,8,9,10,11,12], autophagy, and endoplasmic reticulum stress [13]; however, its effect on migration has rarely been reported.

Migration inhibitory effects of WFA against cancer cells had been reported recently [14,15]. For example, WFA exhibits G2/M cell cycle arrest, apoptosis, and antiproliferation, as well as migration inhibition in gastric cancer AGS cells [14]. However, its migration inhibitory effects were based on wound healing and invasive assays at >10 μM and >1 μM WFA, where the IC_50_ value for WFA in AGS cells was 0.75 μM [14]. WFA also showed antiproliferative effects against breast cancer cells (MDA-MB-241) and could exhibit migration inhibitory effect using the concentration of IC_50_ value for WFA (12 μM) [15]. These migration inhibitory effects of WFA against cancer cells were based on high cytotoxic concentrations. The migration modulating effect of low concentration of WFA with low or no cytotoxicity warrants for detailed investigation.

To date, the migration inhibitory effects of WFA against oral cancer cells had rarely been investigated. Since ROS is a vital factor for cell migration regulation [16], the migration inhibitory effects of low concentration of WFA, as well as the role of WFA-generated ROS in regulating oral cancer cell migration warrants detailed investigation. Accordingly, the aim of this study is to evaluate the migration regulation of low concentration WFA and explore the involvement of oxidative stress in the migration-modulating mechanisms in oral cancer cells.

## 2. Materials and Methods

### 2.1. Cell Culture and Reagents

Ca9-22 oral cancer cell line (Japanese Collection of Research Bioresources Cell Bank; JCRB) were incubated in Dulbecco’s Modified Eagle Medium (DMEM)/Nutrient Mixture F-12 containing 10% bovine serum and penicillin/streptomycin (Gibco, Grand Island, NY, USA), as described previously [17]. WFA and the antioxidant *N*-acetylcysteine (NAC) [18,19] were purchased from Selleckchem.com (Houston, TX, USA) and Sigma-Aldrich (St. Louis, MO, USA).

### 2.2. Cell Viability

Cell viability was determined through mitochondrial enzyme activity detection using MTS assay (Promega Corporation, Madison, WI, USA) as described previously [20].

### 2.3. ROS Flow Cytometry

Cellular ROS content was detected by Accuri C6 flow cytometer (BD Biosciences; Franklin Lakes, NJ, USA) using ROS interacting dye 2′,7′-Dichlorodihydrofluorescein diacetate (DCFH-DA) (Sigma-Aldrich, St. Louis, MO, USA) [21] under the following conditions: 10 μM, 37 °C for 30 min.

### 2.4. Wound Healing Assay

Wound healing assay was used to detect 2D migration ability as described previously [22,23]. The non-migrated cell-free area for vehicle, NAC, WFA, and NAC + WFA (NAC pretreatment and WFA posttreatment) in oral cancer cells were measured using the free software “TScratch” (https://www.cse-lab.ethz.ch/software/).

### 2.5. Cellular 3D Migration and Invasion Assays 

Three-dimensional migration ability was detected using 8 μm pore transwell chambers (Greiner Bio-One; Vilvoorde, Belgium). Three-dimensional invasion ability was detected using 0.5% Matrigel (BD Matrigel Basement Membrane Matrix, BD Biosciences, Bedford, MA, USA) topped transwell chambers. For these two assays, cells were plated under serum-free medium in the transwell top chambers, which were soaked in 10% FBS-containing medium with vehicle, NAC, WFA, and NAC + WFA for 21 h in the bottom chamber. Other detailed steps were described previously [23]. Finally, the 3D migration and invasion abilities were analyzed using Image J software.

### 2.6. Zymography for Matrix Metalloproteinase (MMP)-2 and MMP-9 Activities

Cell invasion ability were proportional to the MMP-2 and MMP-9 activities [24], which were detected using zymography analysis. Cells were seeded overnight, washed with 1X PBS, and treated with vehicle, NAC, WFA, and NAC + WFA in serum-free medium for 48 h. The conditioned medium used for gelatin zymography was described previously [23]. Gelatinase-based MMP-2 and MMP-9 activities were measured by the area of clear zone using Image J software.

### 2.7. Quantitative RT-PCR (qRT-PCR) for Antioxidant-Associated Genes

Total RNA, prepared by Trizol reagent (Invitrogen, Carlsbad, CA, USA), was reverse- transcribed to cDNA using the OmniScript RT kit (Qiagen, Valencia, CA, USA) as described previously [25]. qRT-PCR was performed by iQ SYBR Green Supermix (Bio-Rad Laboratories, Hercules, CA, USA) using a MyiQ real-time machine (Bio-Rad). Touch-down PCR program [26] was used for the antioxidant-associated genes [27], including nuclear factor erythroid 2-like 2 (*NFE2L2*), glutathione-disulfide reductase (*GSR*), glutamate-cysteine ligase catalytic subunit (*GCLC*), glutathione peroxidase 1 (*GPX1*), thioredoxin (*TXN*), catalase (*CAT*), superoxide dismutase 1 (*SOD1*), heme oxygenase 1 (*HMOX1*), NAD(P)H quinone dehydrogenase 1 (*NQO1*), and *GAPDH*. Their primer and PCR amplicon information are provided in Table 1. The comparative method (2–ΔΔCt) was used for analyzing relative mRNA expression (fold activation) [28].

### 2.8. Western Blotting for Mitogen-Activated Protein Kinase (MAPK) Expressions

Total protein (45 μg) was electrophoresed by 10% SDS-PAGE. After PVDF transferring and blocking, primary antibodies recognized extracellular-signal-regulated kinase 1/2 (ERK1/2), c-Jun N-terminal kinase 1/2 (JNK 1/2), p38 (MAPK Family Antibody Sampler Kit; #9926, Cell Signaling Technology, Inc., Danvers, MA, USA), and their phosphorylated forms (Phospho-MAPK Family Antibody Sampler Kit; #9910, Cell Signaling Technology, Inc., Danvers, MA, USA) as well as GAPDH (#GTX627408; GeneTex International Corp.; Hsinchu, Taiwan) were used and other detailed steps were described previously [23]. The band intensity was analyzed using Image J software.

### 2.9. Statistical Analysis

Multiple comparisons were analyzed using the Tukey HSD test (JMP13; SAS Institute, Cary, NC, USA). Treatments without the same letter characters show a significant difference.

## 3. Results

### 3.1. Identification of the Optimal Concentrations of WFA for Oral Cancer Cell Migration Assay

**Figure 1:**
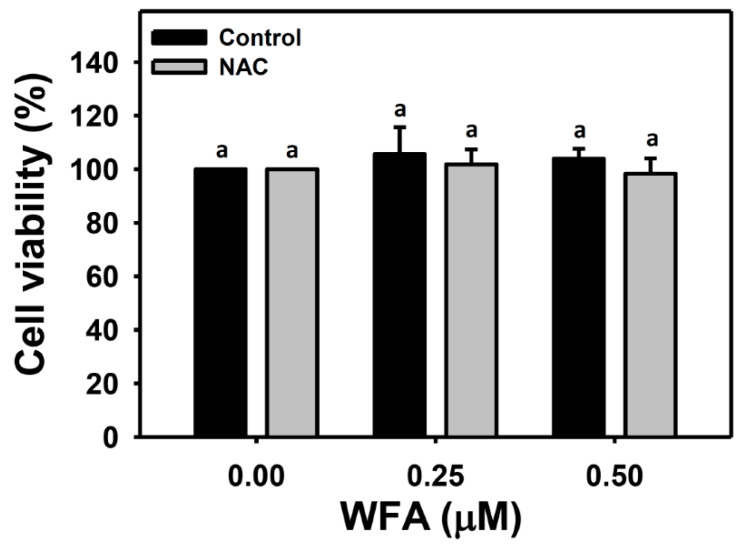
Viability of low concentration of Withaferin A (WFA) treatment in oral cancer cells. Oral cancer cells (Ca9-22) were pretreated with or without *N*-acetylcysteine (NAC) (2 mM, 1 h) and post-treated with different concentrations of WFA for 24 h. For multiple comparison, treatments with the same letter character show nonsignificant difference. Data, mean ± SD (*n* = 3).

In the MTS assay (Figure 1), oral cancer cells (Ca9-22) were treated with 0, 0.25, and 0.5 μM of WFA for 24 h with or without NAC pretreatment (2 mM, 1 h). Neither the WFA nor the NAC + WFA (NAC pretreatment and WFA posttreatment) affect the viability of Ca9-22 cells. This result suggests that WFA under 0.5 μM in the single treatment (WFA) or the combined treatment (NAC + WFA) both exhibited no cytotoxic to oral cancer cells (>95% viability). These concentrations were chosen for the following migration related experiments.

### 3.2. ROS Generation of Oral Cancer Ca9-22 Cells at Low Concentrations of WFA

**Figure 2:**
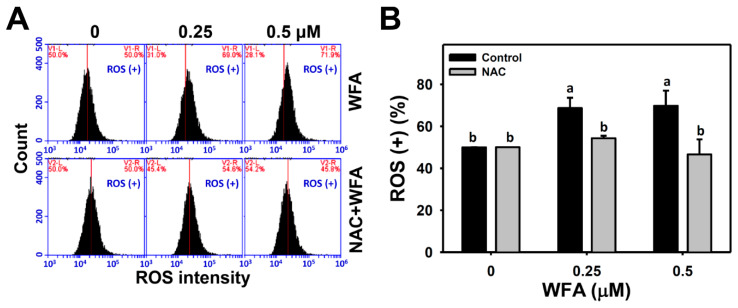
ROS generation effects of low concentrations of WFA in oral cancer cells. (**A**) ROS patterns of Ca9-22 cells after NAC and/or WFA treatments. Cells were pretreated with or without NAC (2 mM, 1 h) and post-treated with different concentrations of WFA for 24 h, i.e., NAC + WFA vs. WFA. ROS-positive population is marked as ROS (+). (**B**) Statistics of ROS change in Figure 2A. For multiple comparison, treatments without the same labels (a,b) indicate the significant difference. *p* < 0.05~0.001. Data, mean ± SD (*n* = 3).

Figure 2A presented ROS patterns of Ca9-22 cells after NAC and/or WFA treatment. The ROS (+) (%) of Ca9-22 cells after low concentrations of WFA treatments were higher than those of the control, whereas this ROS generation was suppressed by NAC pretreatment (Figure 2B). Therefore, low concentrations of WFA triggered moderate ROS generation in oral cancer Ca9-22 cells.

### 3.3. 2D Migration of Oral Cancer Ca9-22 Cells at Low Concentrations of WFA

**Figure 3:**
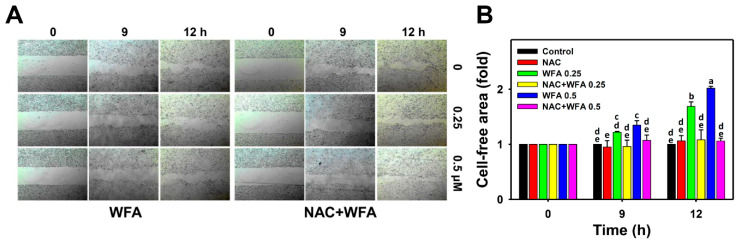
Two-dimensional anti-migration effects of low concentrations of WFA in oral cancer cells. (**A**) Two-dimensional migration (wound healing) images of Ca9-22 cells after NAC and/or WFA treatments. Cells were pretreated with or without NAC (2 mM, 1 h) and post-treated with different concentrations of WFA for 0, 9 and 12 h. (**B**) Statistics of 2D migration change in Figure 3A. For multiple comparison, treatments without the same labels (a–e) indicate the significant difference. *p* < 0.05~0.0001. Data, mean ± SD (*n* = 3).

Figure 3A demonstrated the wound healing patterns of Ca9-22 cells after NAC and/or WFA treatments. Figure 3B showed that the cell-free area (%) of Ca9-22 cells after low concentrations of WFA treatments was greater than that of the untreated control over time. In contrast, this WFA-induced increase of cell-free area (%) was suppressed by NAC pretreatment. Therefore, low concentrations of WFA triggered 2D migration inhibition in Ca9-22 cells.

### 3.4. 3D Migration and Invasion Changes in Oral Cancer Ca9-22 Cells at Low Concentrations of WFA

**Figure 4:**
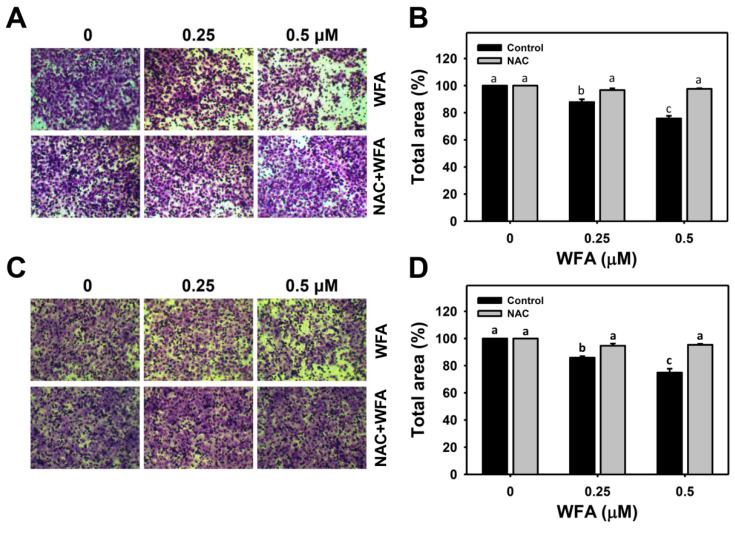
Three-dimensional anti-migration and -invasion effects of low concentrations of WFA in oral cancer cells. (**A**,**C**) 3D migration and invasion images of Ca9-22 cells after NAC and/or WFA treatments. Cells were pretreated with or without NAC (2 mM, 1 h) and post-treated with different concentrations of WFA for 21 h. (**B**,**D**) Statistics of 3D migration and invasion changes in Figure 4A,B. For multiple comparison, treatments without the same labels (a–c) indicate the significant difference. *p* < 0.001~0.0001 (**B**) and *p* < 0.01~0.001 (**D**). Data, mean ± SD (*n* = 3).

To further confirm the 2D migration inhibitory effect of WFA, the 3D migration and invasion assays of Ca9-22 cells were performed (Figure 4A,C, respectively). Figure 4B,D showed that low concentrations of WFA suppressed transwell migration and the Matrigel invasion abilities of Ca9-22 cells in a dose-response manner. In contrast, the WFA-induced 3D migration inhibition and invasion were suppressed by NAC pretreatment. Therefore, low concentrations of WFA triggers inhibitory 3D migration and invasion in Ca9-22 cells.

### 3.5. MMP-2 and MMP-9 Zymography of Oral Cancer Ca9-22 Cells at Low Concentrations of WFA

**Figure 5:**
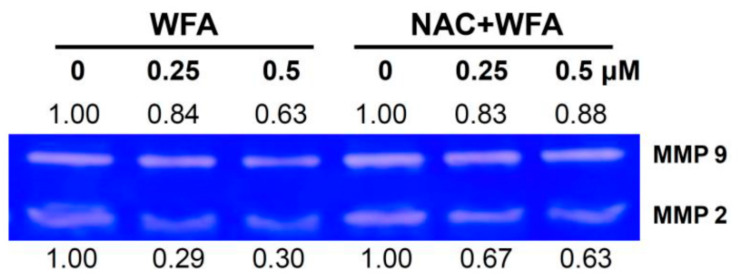
MMP-2 and MMP-9 activities of low concentrations of WFA in oral cancer cells. Zymography-detecting MMP-2 and MMP-9 activities in Ca9-22 cells after NAC and/or WFA treatments. Cells were pretreated with or without NAC (2 mM, 1 h) and post-treated with different concentrations of WFA for 48 h. Similar experiments were repeated 3 times.

MMP-2 and MMP-9 activities were proportional to the cell invasion ability [32]. To detect MMP-2 and MMP-9 activities after low concentrations of WFA treatment, a zymography assay was performed. Figure 5 demonstrated the clear zone pattern of MMP-2 and MMP-9 in Ca9-22 cells after NAC and/or WFA treatment. It showed that the MMP-2 and MMP-9 activities of Ca9-22 cells were decreased after WFA treatment. In contrast, these WFA-induced inhibitions of MMP-2 and MMP-9 activities were suppressed by NAC pretreatment. Therefore, low concentrations of WFA triggers inhibition of MMP-2 and MMP-9 activities in Ca9-22 cells.

### 3.6. Antioxidant Gene Expressions of Oral Cancer Ca9-22 Cells at Low Concentrations of WFA

**Figure 6:**
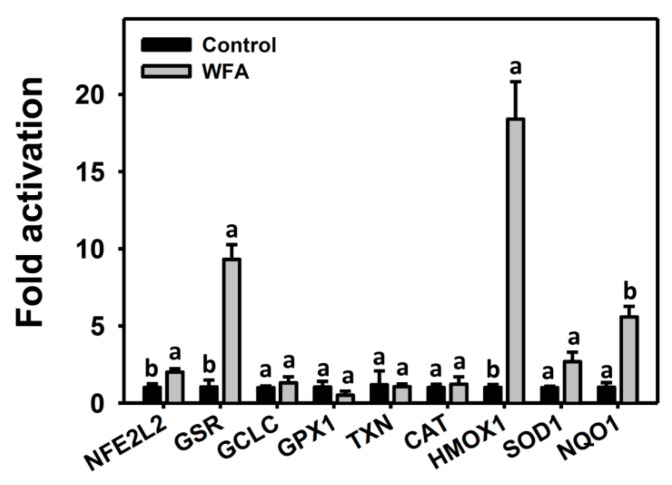
mRNA expressions of antioxidant genes of low concentrations of WFA in oral cancer cells. Cells were treated with or without 0.5 μM of WFA for 24 h. Treatments (control vs. WFA) without the same labels (a,b) indicate the significant difference. *p* < 0.05~0.01. Data, mean ± SD (*n* = 2).

Under oxidative stress, ROS may activate antioxidant pathways [33,34]. Since moderate ROS is induced by low concentrations of WFA, the mRNA expressions of antioxidant genes [27], including *NFE2L2*, *GSR*, *GCLC*, *GPX1*, *TXN*, *CAT*, *SOD1*, *HMOX1*, and *NQO1*, were examined. Figure 6 showed that low concentrations of WFA significantly induced mRNA expressions of *NFE2L2*, *HMOX1*, *GSR*, and *NQO1* genes while expressions of other genes were not significantly affected. Therefore, low concentrations of WFA triggers some antioxidant signaling in Ca9-22 cells.

### 3.7. Mitogen-Activated Protein Kinase (MAPK) Expressions of Oral Cancer Ca9-22 Cells at Low Concentrations of WFA

**Figure 7:**
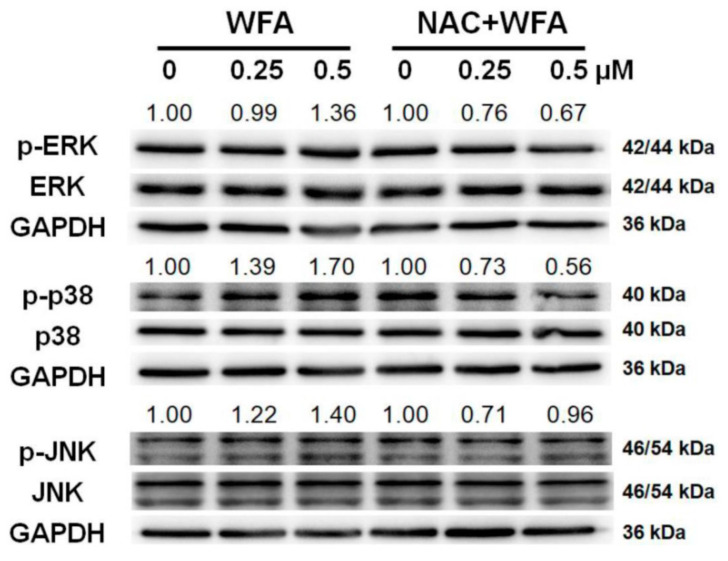
MAPK changes of low concentrations of WFA in oral cancer cells. Ca9-22 cells were pretreated with or without NAC (2 mM, 1 h) and post-treated with different concentrations of WFA for 24 h. ERK1/2, JNK1/2, p38, p-ERK1/2, p-JNK1/2, and p-p38 expressions were detected by Western blotting. The intensity ratio for each p-MAPK expression was adjusted to its matched MAPK and GAPDH intensities. Similar experiments were repeated 3 times.

To further detect the potential upstream antioxidant signaling in oral cancer cells after low concentrations of WFA treatment, the activation of three members of MAPK, including ERK, JNK, and p38 MAPK was examined. Figure 7 showed that WFA induced phosphorylation of three MAPK members, i.e., p-ERK1/2, p-JNK1/2, and p-p38. In contrast, these WFA-induced MAPK phosphorylations were suppressed by NAC pretreatment. Therefore, low concentrations of WFA triggers MAPK phosphorylations in Ca9-22 cells.

## 4. Discussion

Previously, we discovered that high cytotoxic concentration of WFA, which was larger than IC_50_, selectively killed oral cancer cells but rarely damaged normal oral cells [12], i.e., IC_50_ value of WFA in oral cancer Ca9-22 cells is 3 µM at 24 h MTS assay. In the current study, we focus on the evaluation of the migration regulating effects of low concentration (within 0.5 μM) of WFA in oral cancer Ca9-22 cells, which show 95% viability. This low concentration of WFA inhibits 2D/3D migration, 3D invasion, MMP-2 and MMP-9 activities, whereas it induces ROS generation, antioxidant related gene mRNA expressions and MAPK phosphorylation. The detailed mechanisms for low concentration of WFA inducing inhibition of migration and invasion are discussed below.

### 4.1. Low Cytotoxic Concentration of Drugs Is Suitable for Migration Study

The standard criteria for studying the migration effect of drugs is based on measurements using low cytotoxic concentrations [35,36,37]. With a high cytotoxic concentration (higher than IC_50_), WFA had been reported to show migration inhibitory effects against gastric [14] and breast [15] cancer cells, though it may be attributed to apoptosis and cell death. Alternatively, low concentration of WFA with no cytotoxicity avoided side effect of cell death and provided a clear observation for migration response in the current study.

### 4.2. MMP-2 and MMP-9 Activity Changes in WFA-Treated Oral Cancer Cells

MMP-2 and MMP-9 are important mediators for cell migration, invasion, and metastasis in carcinogenesis [24]. A WFA-derived compound such as 3-azido WFA inhibits MMP-2 activity and migration of prostate PC-3 and cervical HeLa cancer cells [38]. Low concentration of WFA (>95% viability) inhibits MMP-9 activity of cervical Caski and liver SK-Hep-1 cancer cells by downregulating Akt phosphorylation [39]. 

In agreement with the inhibitory effect on MMP-9 activity [39], we further found that low concentration of WFA (>95% viability) exhibits inhibitory effects on MMP-2 activity in oral cancer Ca9-22 cells. Accordingly, WFA inhibits migration of oral cancer cells by inactivating MMP-2 and MMP-9. Moreover, MMP-2 and MMP-9 are overexpressed in the biopsy specimens of oral squamous cell carcinoma compared to the adjacent normal tissues [40,41]. Therefore, a low concentration of WFA has the potential to inhibit the MMP-2 and MMP-9 activities in order to inhibit migration or metastasis of oral cancer cells.

### 4.3. ROS Changes in WFA-Treated Oral Cancer Cells

As mentioned above, WFA exhibits a concentration-effect on apoptosis and migration, i.e., high concentration of WFA induces apoptosis while low concentration of WFA inhibits migration. Our previous study [12] demonstrated that the cytotoxic concentrations (>IC_50_) of WFA induced 90% (+) ROS in oral cancer Ca9-22 cells. In the current study, the low concentration (>95% viability) of WFA induces 70% (+) ROS generation in Ca9-22 cells. It is possible that low concentration of WFA induces a ROS level lower than the redox threshold and leads to cell survival with inhibitory migration. In contrast, high cytotoxic concentration of WFA induces a ROS level higher than the redox threshold and leads to apoptosis and cell death. Accordingly, the differential ROS induction by WFA may lead to distinct fate of oral cancer cells, i.e., migration inhibition or inducible apoptosis. 

### 4.4. Antioxidant Genes Changes in WFA-Treated Oral Cancer Cells

In cancer cells, ROS overproduction is counterbalanced by overexpression of antioxidant activity for redox homeostasis [42]. Moreover, antioxidant genes have the potential to regulate cellular migration. For example, knockdown of *HMOX1* and/or *NFE2L2* reversed the migration inhibitory effect of semaphorin 6A (SEMA6A) and the SEMA6A-driven downregulation of MMP-9 [43]. Knockdown of *NQO1* increases the invasion of human cutaneous squamous cancer SCC12 and SCC13 cells but it is reverted by *NQO1* overexpression [44]. Consistently, we found that low concentrations of WFA induced mRNA expressions of *NFE2L2*, *HMOX1*, and *NQO1* genes, which may lead to inhibitory migration of oral cancer cells.

### 4.5. MAPK Changes in WFA-Treated Oral Cancer Cells

As mentioned above, both high [12] and low (the current study) concentrations of WFA induced ROS. Moreover, ROS can regulate MAPK signaling [45], which is associated with tumor cell invasion [46]. Cytotoxic concentration of WFA induces apoptosis by phosphorylating p38 and ERK1/2 in leukemic [47] and glioblastomas cells [48], respectively. Similarly, we found that low concentration of WFA induces mild phosphorylation for ERK, JNK, and p38 MAPK.

### 4.6. The Role of ROS in Low Concentration of WFA Induced Migration Changes and Signaling in Oral Cancer Cells

Under low concentration of WFA, the changes of ROS generation, 2D migration, 3D migration/invasion, MMP-2/MMP-9 activities, antioxidant gene expression, and MAPK phosphorylation are reverted by NAC pretreatment. These results indicate that a low concentration of WFA inhibits migration and induces antioxidant signaling in a ROS-dependent manner in oral cancer cells.

## 5. Conclusions

Our study focuses on low concentrations of WFA to evaluate its inhibitory effects on migration and invasion in oral cancer Ca9-22 cells. Under low concentrations of WFA, Ca9-22 cells are grown with high viability and retained anti-migration and anti-invasion. Mechanically, this safe treatment of WFA inhibits MMP-2 and MMP-9 activities and induces antioxidant gene expression as well as MAPK activation in oral cancer cells. All these inhibitory migration changes and mechanisms after WFA treatment were suppressed by NAC pretreatment, suggesting that ROS plays an important role in WFA induced inhibitory migration in oral cancer cells. In conclusion, we provide here the first finding that supports low concentration of WFA could be a potent inhibitor for metastasis in oral cancer therapy.

## Figures and Tables

**Table 1:** Primer information for antioxidant-associated genes *.

Genes	Forward Primers (5′→3′)	Reverse Primers (5′→3′)	Length
*TXN*	GAAGCAGATCGAGAGCAAGACTG	GCTCCAGAAAATTCACCCACCT	270 bp
*GSR*	GTTCTCCCAGGTCAAGGAGGTTAA	CCAGCAGCTATTGCAACTGGAGT	297 bp
*CAT*	ATGCAGGACAATCAGGGTGGT	CCTCAGTGAAGTTCTTGACCGCT	274 bp
*SOD1*	AGGGCATCATCAATTTCGAGC [29]	CCCAAGTCTCCAACATGCCTC	211 bp
*HMOX1*	CCTTCTTCACCTTCCCCAACAT	GGCAGAATCTTGCACTTTGTTGC	251 bp
*NFE2L2*	GATCTGCCAACTACTCCCAGGTT	CTGTAACTCAGGAATGGATAATAGCTCC	302 bp
*NQO1*	GAAGGACCCTGCGAACTTTCAGTA	GAAAGCACTGCCTTCTTACTCCG	258 bp
*GCLC*	ACAAGCACCCTCGCTTCAGTACC	CTGCAGGCTTGGAATGTCACCT	232 bp
*GPX1*	AACCAGTTTGGGCATCAGGAG	AGTTCCAGGCAACATCGTTGC	256 bp
*GAPDH*	CCTCAACTACATGGTTTACATGTTCC [30]	CAAATGAGCCCCAGCCTTCT [31]	220 bp

* Primers without reference were designed in this study.

## Abbreviations

WFA: withaferin A; ROS, reactive oxygen species; MMP, matrix metalloproteinase; *NFE2L2*, nuclear factor, erythroid 2-like 2; *HMOX1*, heme oxygenase 1; *GSR*, glutathione-disulfide reductase; *NQO1*, NAD(P)H quinone dehydrogenase 1; MAPK, mitogen-activated protein kinase; ERK1/2, extracellular signal-regulated kinases 1/2; JNK, c-Jun N-terminal kinase; NAC, *N*-acetylcysteine; DCFH-DA, 2′,7′-Dichlorodihydrofluorescein diacetate; qRT-PCR, quantitative RT-PCR; *NFE2L2*, nuclear factor erythroid 2-like 2; *GCLC*, glutamate-cysteine ligase catalytic subunit; *GPX1*, glutathione peroxidase 1; *TXN*, thioredoxin; *CAT*, catalase; *SOD1*, superoxide dismutase 1.
